# Retraction: Comparison of acupuncture and other drugs for chronic constipation: A network meta-analysis

**DOI:** 10.1371/journal.pone.0201274

**Published:** 2018-07-19

**Authors:** The PLOS ONE Editors

After publication of this article [1], concerns were raised about the scientific validity of the meta-analysis and whether it provided a rigorous and accurate assessment of published clinical studies on the efficacy of acupuncture or drug-based interventions for improving chronic constipation. The *PLOS ONE* Editors re-assessed the article in collaboration with a member of our Editorial Board and noted several concerns including the following:

- Acupuncture and related terms are not mentioned in the literature search terms, there are no listed inclusion or exclusion criteria related to acupuncture, and the outcome measures were not clearly defined in terms of reproducible clinical measures.
- The study included acupuncture and electroacupuncture studies, though this was not clearly discussed or reported in the Title, Methods, or Results.
- In the "Routine paired meta-analysis" section, both acupuncture and sham acupuncture groups were reported as showing improvement in symptoms compared with placebo. This finding and its implications for the conclusions of the article were not discussed clearly.
- Several included studies did not meet the reported inclusion criteria requiring that studies use adult participants and assess treatments of >2 weeks in duration.
- Data extraction errors were identified by comparing the dataset used in the meta-analysis (S1 Table) with details reported in the original research articles. Errors included aspects of the study design such as the experimental groups included in the study, the number of study arms in the trial, number of participants, and treatment duration. There are also several errors in the Reference list.
- With regard to side effects, 22 out of 40 studies were noted as having reported side effects. It was not made clear whether side effects were assessed as outcome measures for the other 18 studies, i.e. did the authors collect data clarifying that there were no side effects or was this outcome measure not assessed or reported in the original article. Without this clarification the conclusion comparing side effect frequencies is not well supported.
- The network geometry presented in Fig 5 is not correct and misrepresents some of the study designs, for example showing two-arm studies as three-arm studies.
- The overall results of the meta-analysis are strongly reliant on the evidence comparing acupuncture versus lactulose treatment. Several of the trials that assessed this comparison were poorly reported, and the meta-analysis dataset pertaining to these trials contained data extraction errors. Furthermore, potential bias in studies assessing lactulose efficacy in acupuncture trials versus lactulose efficacy in other trials was not sufficiently addressed.

While some of the above issues could be addressed with additional clarifications and corrections to the text, the concerns about study inclusion, the accuracy with which the primary studies’ research designs and data were represented in the meta-analysis, and the reporting quality of included studies directly impact the validity and accuracy of the dataset underlying the meta-analysis. As a consequence, we consider that the overall conclusions of the study are not reliable. In light of these issues, the *PLOS ONE* Editors retract the article. We apologize that these issues were not adequately addressed during pre-publication peer review.

LZ disagreed with the retraction. YM and XD did not respond.
